# Oxygen-dependent proteolysis regulates the stability of angiosperm polycomb repressive complex 2 subunit VERNALIZATION 2

**DOI:** 10.1038/s41467-018-07875-7

**Published:** 2018-12-21

**Authors:** Daniel J. Gibbs, Hannah M. Tedds, Anne-Marie Labandera, Mark Bailey, Mark D. White, Sjon Hartman, Colleen Sprigg, Sophie L. Mogg, Rory Osborne, Charlene Dambire, Tinne Boeckx, Zachary Paling, Laurentius A. C. J. Voesenek, Emily Flashman, Michael J. Holdsworth

**Affiliations:** 1 School of Biosciences, University of Birmingham, Edgbaston, B15 2TT UK; 2 Chemistry Research Laboratory, University of Oxford, 12 Mansfield Road, Oxford, OX1 3TA UK; 3 Plant Ecophysiology, Institute of Environmental Biology, Faculty of Science, Utrecht University, Padualaan 8, Utrecht, 3584 CH The Netherlands; 4 School of Biosciences, University of Nottingham, Loughborough, LE12 5RD UK

## Abstract

The polycomb repressive complex 2 (PRC2) regulates epigenetic gene repression in eukaryotes. Mechanisms controlling its developmental specificity and signal-responsiveness are poorly understood. Here, we identify an oxygen-sensitive N-terminal (N-) degron in the plant PRC2 subunit VERNALIZATION(VRN) 2, a homolog of animal Su(z)12, that promotes its degradation via the N-end rule pathway. We provide evidence that this N-degron arose early during angiosperm evolution via gene duplication and N-terminal truncation, facilitating expansion of PRC2 function in flowering plants. We show that proteolysis via the N-end rule pathway prevents ectopic VRN2 accumulation, and that hypoxia and long-term cold exposure lead to increased VRN2 abundance, which we propose may be due to inhibition of VRN2 turnover via its N-degron. Furthermore, we identify an overlap in the transcriptional responses to hypoxia and prolonged cold, and show that VRN2 promotes tolerance to hypoxia. Our work reveals a mechanism for post-translational regulation of VRN2 stability that could potentially link environmental inputs to the epigenetic control of plant development.

## Abstract (web summary)

VRN2 is a Polycomb Repressive Complex 2 subunit, best known as a regulator of vernalization that accumulates during prolonged cold. Here Gibbs *et al*. show that VRN2 is degraded via the N-end rule pathway, which  prevents ectopic accumulation of VRN2 in the absence of appropriate environmental stimuli.

## Introduction

Polycomb group (PcG) proteins are essential regulators of gene expression in eukaryotes, functioning as multiprotein complexes to establish epigenetically silenced states on their gene targets^1–3^. One of the best characterized of these complexes is the polycomb repressive complex 2 (PRC2), which catalyses the deposition of the repressive histone H3 lysine 27 trimethylation (H3K27me3) mark on chromatin^4,5^. This modification is mitotically stable and therefore acts as a long-term (yet reversible) suppressor of gene transcription. PRC2 activity controls cell identity, developmental transitions and the establishment of environmental memory across kingdoms^4,5^. Although many PRC2 functions and gene targets have been identified, mechanisms underpinning signal perception and transduction via this complex, as well as its developmental specificity, are still poorly understood.

The canonical PRC2 comprises four subunits: ENHANCER OF ZESTE [E(z)], SUPRESSOR OF ZESTE 12 [Su(z)12], EXTRA SEX COMBS (Esc) and p55^2,4,5^. E(z) is the catalytic component with histone-methyltransferase (HMTase) activity, whilst Su(z)12 and Esc are both required for complex integrity and facilitating methylation by E(z)^2,6^. A wide range of context-specific binding partners also contribute to specificity, efficiency and robustness of PRC2 activity^2,5^. In contrast to animals, plants encode multiple copies of PRC2 subunits^5,7^, allowing the formation of different complexes with distinct functions, although knowledge of how and why these components diverged during evolution to acquire new roles is limited. In *Arabidopsis thaliana*, PRC2 complexes are classified depending on which one of three Su(z)12-like subunits they recruit: FERTILIZATION INDEPENDENT 2 (FIS2), EMBRYONIC FLOWER 2 (EMF2) or VERNALIZATION 2 (VRN2)^5,7,8^. FIS2-PRC2 inhibits seed development in the absence of fertilization^9^, whilst EMF2-PRC2 promotes vegetative growth through suppressing flowering^10^. VRN2-PRC2 has several developmental functions^11–13^, but is best known as a key regulator of vernalization, the epigenetic process by which long-term cold exposure promotes the transition from vegetative to reproductive development^14^. During vernalization, VRN2-PRC2 methylates and silences the floral repressor gene *FLOWERING LOCUS C* (*FLC*), thereby encoding a memory of cold that permits flowering once warm temperatures return. A molecular mechanism regulating the cold-triggered accumulation^15^ of VRN2 required for this process is not known.

**Fig. 1:**
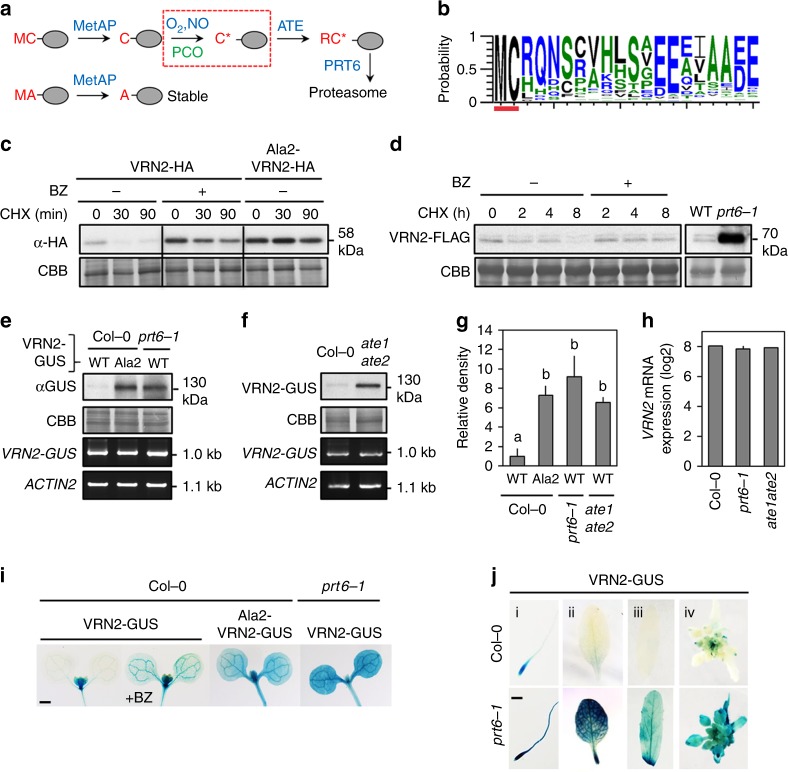
VRN2 is a substrate of the Arg/N-end rule pathway of proteolysis. **a** N-terminal processing events leading to degradation of Met-Cys- (MC)-proteins via the Arg(R)/N-end rule pathway: MetAP methionine aminopeptidase, PCO plant cysteine oxidase (specific to plants), ATE arginyl transferase, PRT6 proteolysis 6, C* Cys-sulfinic acid (oxidised Cys); red box is gas-dependent step. **b** Probability plot of AA by position in VRN2-like sequences derived from sequenced angiosperms following MUSCLE alignment, highlighting the conserved MC N-terminus (red bar). See Supplementary Data 2 for sequence data. **c** In vitro cycloheximide (CHX) chase of WT and mutant (Ala2) VRN2-HA (±bortezomib; BZ). CBB Coomassie brilliant blue (showing equal loading). **d** CHX-chase of VRN2-FLAG in 7-day-old seedlings ± BZ treatment, and steady state levels in *prt6-1* vs WT. **e**, **f** Protein and mRNA levels of WT and mutant (Ala2) VRN2-GUS in Col-0, *prt6-1 and ate1 ate2* seedlings. **g** VRN2-GUS protein accumulation in Col-0, *prt6-1* and *ate1 ate2* shown as relative density. Data is averaged from western blots for three independent lines for each transgene (see also Supplementary Figure 1a and b). Letters indicate one-way ANOVA; Tukey’s test. **h** *VRN2* mRNA expression levels in Col-0, *prt6-1* and *ate1 ate2*, taken from published microarray data^19^. **i** Histochemical staining of 7-day-old Col-0 or *prt6-1* seedlings expressing WT or mutant (Ala2) VRN2-GUS ± BZ. Lines are the same as those presented in (**e**) and (**f**). Scale bar 500 μm. See also supplementary Figure 2a for biological reps. **j** Histochemical staining of (i) 7-day-old seedling primary root tip, (ii) rosette leaf, (iii) cauline leaf and (iv) inflorescence of Col-0 and *prt6-1* lines expressing WT VRN2-GUS. Scale bar in (i), 500 μm. Source data are provided as a Source Data file

The evolutionarily conserved N-end rule pathway regulates protein destruction through the recognition of N-terminal degradation sequences (N-degrons) in target proteins, which promote their ubiquitylation by specific E3 ligases (N-recognins)^16–18^. Several branches of the N-end rule pathway are known, which mediate a broad range of growth, developmental and stress-associated processes across kingdoms (reviewed in refs. ^16,17^). In plants, group VII ETHYLENE RESPONSE FACTOR (ERFVII) transcription factors are Methionine-Cysteine- (Met-Cys-; MC-) initiating substrates of the arginylation (Arg)/N-end rule pathway^19–21^, which are regulated by oxygen (O_2_) and nitric oxide (NO), similar to several REGULATOR OF G PROTEIN SIGNALLING (RGS) proteins in mammals^22,23^. ERFVIIs are targeted for destruction in normoxia via O_2_-dependent oxidation of Nt-Cys, which involves the successive actions of METHIONINE AMINOPEPTIDASES, PLANT CYSTEINE OXIDASES (PCOs)^24,25^, ARGINYL TRANSFERASES (ATEs), and recognition by the N-recognin PROTEOLYSIS6 (PRT6) (summarized in Fig. 1a). NO is also required for degradation^21^, although whether it is involved in Nt-Cys oxidation, or acts indirectly via other factors is currently not known. Under low-O_2_ conditions (hypoxia), Nt-Cys oxidation is limited, leading to increased protein accumulation and function. Thus, ERFVII stability and activity is coupled to O_2_ (and NO) availability, which is important for regulating the survival of flooding stress, other abiotic and biotic stresses, and photomorphogenesis^26–29^. We hypothesized that hypoxia may regulate further aspects of development or environment-response through modulating the stability of other MC-initiating regulatory proteins with different cellular functions.

Here, we identify the plant PRC2 subunit VRN2 as a substrate of the Arg/N-end rule pathway via its conserved N-terminal Cys2 residue. Despite constitutive *VRN2* expression, this post-translational regulation confines VRN2 to the meristems of roots and shoots under non-vernalizing and aerobic growth conditions. We show that both submergence-induced hypoxia and long-term cold exposure lead to enhanced VRN2 accumulation throughout the plant. Cold-exposure induced several transcriptional and post-translational changes typically associated with hypoxia, including ERFVII stabilization and accumulation of hypoxia-related transcripts, suggesting overlap in the cellular response to both conditions. Thus our work identifies the N-end rule pathway as a key determinant of tissue-specific and environment-responsive VRN2-PRC2 activity. Furthermore, phylogenetic and biochemical analyses provide evidence that the N-degron of VRN2 was exposed early in angiosperm evolution through gene duplication and N-terminal truncation of an ancient homolog, which facilitated the expansion of PRC2 function in plants through linking the stability of Su(z)12 to the perception of the environment.

## Results

### VRN2 is a substrate of the Arg/N-end rule pathway

We identified VRN2 as a candidate target N-end rule substrate, due to its MC-initiating N-terminus, which is conserved throughout angiosperms (Fig. 1b). Wild type (WT) VRN2-HA expressed in an in vitro rabbit reticulocyte system (containing functional N-end rule pathway components^19^) was degraded following treatment with the translation inhibitor cycloheximide (CHX), whereas co-treatment with the proteasome inhibitor bortezomib (BZ), or mutation of Cys2 to Alanine (Ala; a stabilizing residue), prevented this turnover (Fig. 1c). VRN2-FLAG^15^ was also unstable in transgenic seedlings, but stabilized by BZ, indicating it is regulated via the 26S proteasome in vivo (Fig. 1d). To test if this regulation is linked to the N-end rule pathway, we introduced *VRN2-FLAG* into *prt6-1* (that lacks PRT6 E3 ligase activity^19^), and observed increased protein accumulation compared to WT (Fig. 1d). Next, we generated a series of transgenic plants expressing WT or mutant (Ala2) VRN2-GUS (β-glucuronidase) fusions driven by approximately 2 kb of the native *VRN2* promoter. Similarly to VRN2-FLAG, we observed accumulation of VRN2-GUS in both *prt6-1* and *ate1 ate*2 (that lacks ATE activity^19^), as well as higher steady-state levels of mutant Ala2-VRN2-GUS protein relative to WT VRN2-GUS in Col-0 (Fig. 1e–g and Supplementary Figure 1a, b). Transgene-specific RT-PCR showed that there were no significant differences in WT-VRN2-GUS or Ala2-VRN2-GUS expression in the different genetic backgrounds, confirming that these changes in abundance relate to post-translational control, and published microarray data^19^ confirmed that endogenous *VRN2* mRNA levels are also similar across genotypes (Fig. 1h). Histochemical staining of 7-day-old seedlings showed that VRN2-GUS in WT is largely confined to the meristematic regions of shoots and roots (Fig. 1i, j and Supplementary Figure 2a, b). In contrast, treatment with BZ, expression in *prt6-1*, or an Ala2 mutation, all led to enhanced stability and a much broader domain of accumulation throughout seedlings and adult plants (Fig. 1i, j and Supplementary Figure 2). This reveals that the N-end rule pathway confines VRN2 accumulation to meristematic tissues, despite constitutive *VRN2* RNA expression^19^ (Supplementary Figure 3).

### VRN2 is regulated by O_2_ and contributes to hypoxia tolerance

**Fig. 2:**
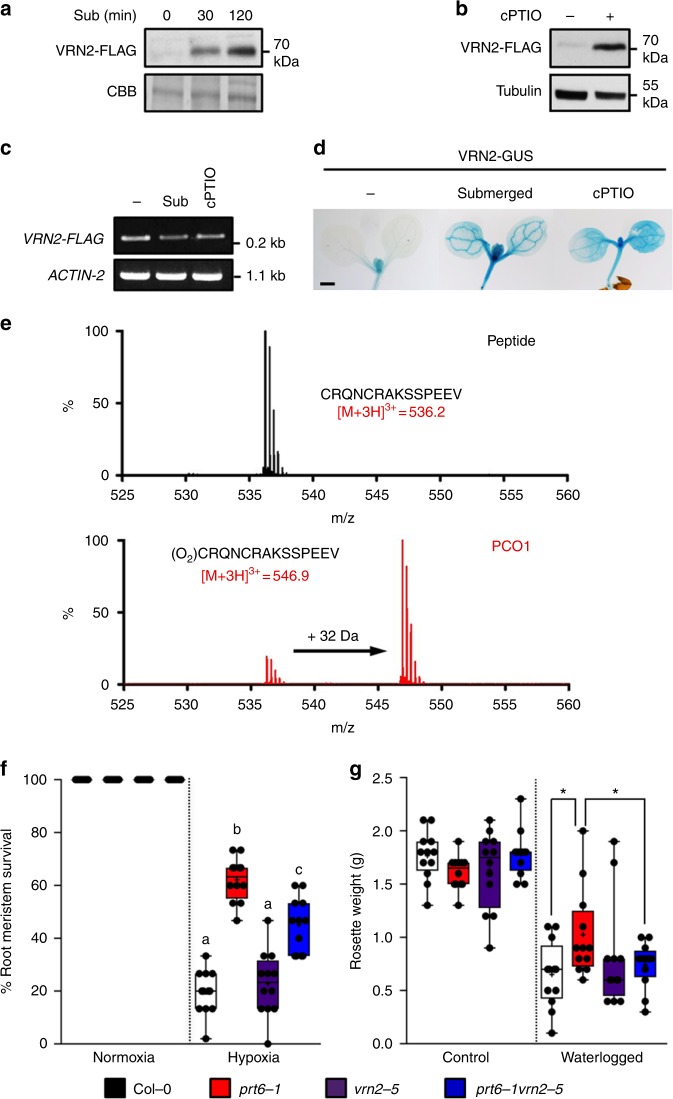
VRN2 is regulated by O_2_ and NO availability and positively regulates hypoxia tolerance. **a** VRN2-FLAG protein accumulation during a submergence time course. **b** VRN2-FLAG protein accumulation ± NO scavenger cPTIO. **c** mRNA levels of *VRN2-FLAG*  ± submergence or cPTIO treatment. **d** Histochemical staining of 7-day-old Col-0 seedlings expressing WT VRN2-GUS ± submergence-induced hypoxia or cPTIO treatment. Scale bar 500 μm. See also Supplementary Figure 2a for biological reps. **e** LC–MS spectra showing the VRN2_2–15_ peptide species identified following incubation with or without recombinant PCO1. A product with a mass increase of +32 Da, indicating O_2_-dependent Cys conversion to Cys-sulfinic acid, was only observed in the presence of PCO1. **f** Root tip survival data for 4-day-old Arabidopsis seedlings in normoxia or following 4 h hypoxia treatment (*n* = 15 × 9 for each genotype). Letters indicate one-way ANOVA; Tukey’s test (*p* < 0.01). **g** Rosette weight of soil-grown plants ±21 days waterlogging treatment (*n* = 12 for each genotype). ANOVA was not significant (*p* < 0.08). Pairwise *t*-tests (shown on graph; Col-0 vs *prt6-1* and *prt6-1* vs *prt6-1 vrn2-5*) were significant (*p < 0.05). Box and whiskers plots show replicates (dots), max and min, 25th to 75th percentiles, median and mean (+). Source data are provided as a Source Data file

Since degradation of VRN2 via the N-end rule pathway is dependent on its Cys2 residue, we examined the influence of O_2_ and NO on VRN2 stability. We observed strong post-translational accumulation of VRN2-FLAG and VRN2-GUS in seedlings exposed to submergence-induced hypoxia or the NO scavenger cPTIO (Fig. 2a–d and Supplementary Figure 2a). We also found that recombinant Arabidopsis PCOs could oxidize in vitro the Nt-Cys residue of a peptide representing the Met1-excised N-terminus of VRN2. Mass shifts of 32 Da were observed in the presence of all 5 PCOs (Fig. 2e and Supplementary Figure 4a), signifying O_2_-dependent Cys-sulfinic acid generation, which is required for subsequent ATE activity and recognition by PRT6^25^. Previous work showed that *prt6-1* has enhanced hypoxia resistance, due to the constitutive accumulation of ERFVIIs^19^. To assess if ectopic VRN2 accumulation in *prt6-1* also contributes to this resilience, we isolated a new null T-DNA allele in Col-0, *vrn2-5* (Supplementary Figure 5), and generated combination mutants for assessing seedling root tip survival following hypoxic stress. Survival of root tips under hypoxia was much greater in *prt6-1* than Col-0, but was significantly reduced in the *prt6-1 vrn2-5* double mutant (Fig. 2f). This double mutant was also more sensitive than *prt6-1* to root waterlogging (Fig. 2g), a distinct but eco-physiologically relevant root hypoxia stress. Collectively, these data define an O_2_-sensitive Cys2 N-degron in VRN2. This N-degron restricts VRN2 accumulation to meristems, which are proposed to be naturally hypoxic due to high metabolic activity and oxygen consumption^30^. VRN2 is stabilized throughout the plant in response to low-O_2_, where our genetic evidence suggests it contributes to hypoxic stress-survival.

### VRN2 abundance is restricted in the absence of cold

**Fig. 3:**
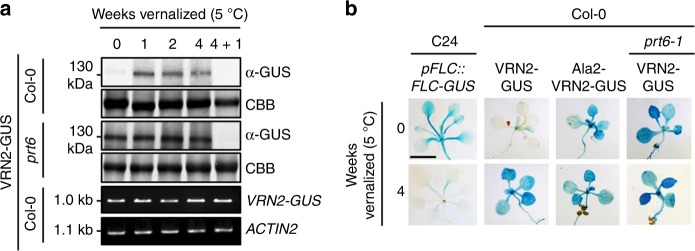
The N-end rule pathway restricts VRN2 accumulation under non-vernalizing conditions. **a** Protein and mRNA levels of WT VRN2-GUS in vernalized (1–4 weeks) Col-0 and *prt6-1* seedlings. 4 + 1 refers to 1 week ‘recovery’ at 22 °C following vernalization. **b** Histochemical staining of seedlings expressing *pFLC::FLC-GUS* (C24 background), and WT or mutant (Ala2) VRN2-GUS in Col-0 and *prt6-1*, ±4 weeks vernalization. Scale bar 5 mm. See also Supplementary Figure 2c for biological reps. Source data are provided as a Source Data file

We next investigated whether the N-end rule pathway regulates the known post-translational accumulation of VRN2 in response to long-term cold exposure, which is required for vernalization^14,15^. VRN2-GUS protein levels increased in Col-0 seedlings after 1 week at 5 °C, without any detectable mRNA changes (Fig. 3a). In contrast, VRN2-GUS was already present at high levels in *prt6-1* at 22 °C, showing only a small increase during cold treatment. This enhanced abundance was maintained throughout subsequent continued cold exposure. The spatial patterning of VRN2-GUS in seedlings after 4 weeks at 5 °C resembled that observed in *prt6-1* (or mutant Ala2-VRN2-GUS in WT) at 22 °C, and was inversely correlated with cold-responsive *pFLC::FLC-GUS*^31^ repression observed in the vernalization-dependent C24 background (Fig. 3b and Supplementary Figure 2c), in accordance with *FLC* being repressed by VRN2-PRC2. A return to 22 °C after 4 weeks of cold exposure led to depletion of VRN2 in both WT and *prt6-1*, indicating the presence of other degron(s) in VRN2 independent of the Arg/N-end rule pathway. These data reveal that the N-end rule pathway limits VRN2 accumulation under non-vernalizing conditions.

### Molecular responses to cold-exposure and hypoxia

**Fig. 4:**
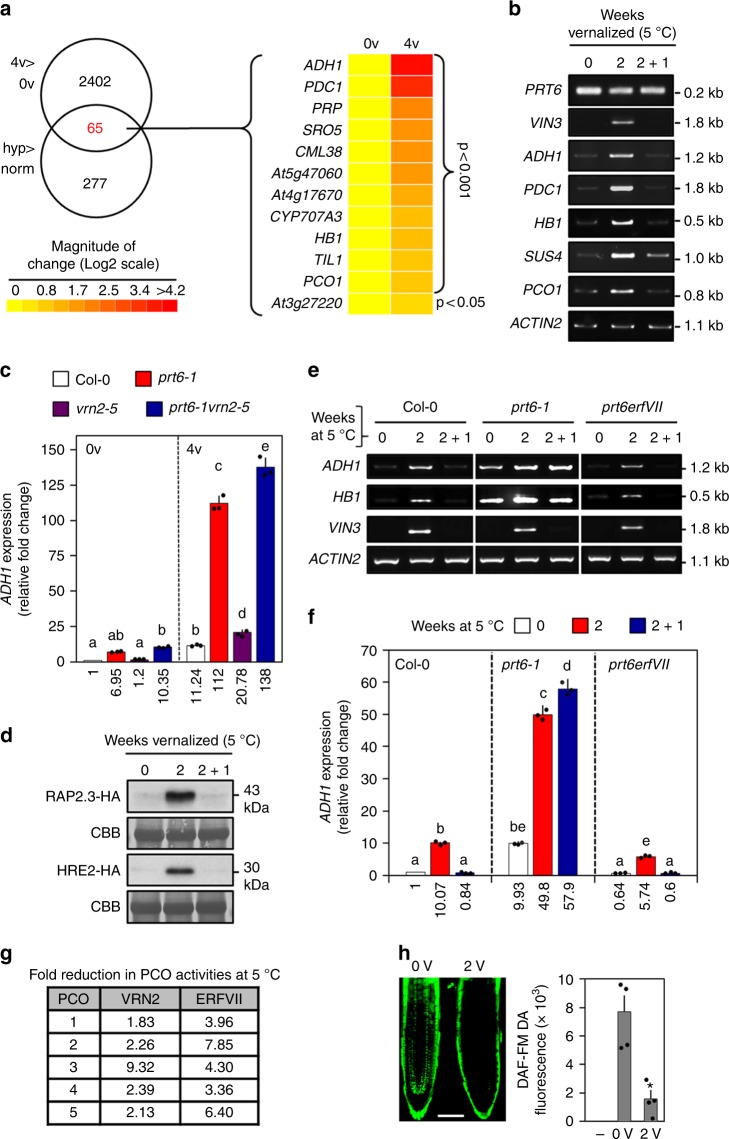
Transcriptional and post-translational responses to cold exposure. **a** Venn diagram showing overlap in upregulated genes between 4-week-vernalized vs non-vernalized (4v > 0v) and hypoxia vs normoxia^21^ (hyp > norm) treated seedlings (*n* = 3 per treatment). Heat map shows Log2 expression levels of 12 core anaerobic genes that are induced in both datasets (for numeric expression values, see Supplementary Data 1). **b** Semi-quantitative RT-PCR analysis of genes in Col-0 seedlings treated±2 weeks vernalization, or 2 weeks vernalization followed by 1 week ‘recovery’ at 22 °C (2 + 1). *VIN3* induction serves as a control for cold exposure. **c** qPCR of *ADH1* levels in Col-0, *prt6-1*, *vrn2-5* and *prt6-1 vrn2-5* seedlings with (4v) or without (0v) 4 weeks cold treatment. Each bar shows the mean of 3 biological reps (dots). Expression values are shown underneath and letters indicate one-way ANOVA; Tukey’s test (*p* < 0.01). **d** Steady state protein levels of the ERFVII transcription factors RAP2.3-HA and HRE2-HA in seedlings vernalized as in (**b**). **e** Semi-quantitative RT-PCR analysis of genes in Col-0, *prt6-1* and *prt6 erfVII* seedlings treated as in (**b**). **f** qPCR of *ADH1* levels in Col-0, *prt6-1* and *prt6 erfVII* seedlings treated as in (**b**). Each bar shows the mean of 3 biological reps (dots). Expression values are shown underneath and letters indicate one-way ANOVA; Tukey’s test (*p* < 0.01). **g** Fold reduction in the activities of PCO1-5 when incubated with VRN2_2–15_ and ERFVII_2–15_ N-terminal peptides at 5 °C relative to 21 °C (see also Supplementary Figure  4b–d). **h** NO levels (measured as DAF-FM fluorescence) in non-vernalized (0v) or 2-week-vernalized (2v) Col-0 roots. Scale bar 50 μm. Each bar shows the mean of 4 biological reps (dots). Error bars are SEM, *p < 0.05 (*t*–test). Source data are provided as a Source Data file

The pattern of VRN2 protein accumulation in response to cold (Fig. 3b) is similar to that observed in response to submergence (Fig. 2d). In both conditions, the accumulation of VRN2 could be determined by reduced degradation via its N-degron. We therefore considered possible mechanism(s) by which long-term cold exposure might promote stabilization of VRN2. Previous studies showed that ALCOHOL DEHYDROGENASE (ADH), a key hypoxia-induced protein, accumulates in cold-treated plants (including Arabidopsis, maize and rice)^32,33^. Therefore, we investigated the potential link between gene-expression regulation in response to long-term cold and hypoxia. Using RNA seq analysis of non-vernalized vs 4-week-vernalized seedlings, we compared cold-induced transcripts with those induced by hypoxia^21^. This revealed a significant overlap in gene expression between the two conditions (*p* < 0.0015; hypergeometric test^21^). Approximately 20% of hypoxia-upregulated genes^21^ were upregulated by long-term cold treatment, including many of the core anaerobic-response genes that are universally induced by low-O_2_^34^ (Fig. 4a, b)_._ Amongst the most highly upregulated transcripts were mRNAs critical for the survival of anaerobiosis (including *ADH1* and *PDC1*), as well as *VIN3*, which interacts with VRN2-PRC2 to promote *FLC* silencing during vernalization^15,35^, and which was previously also shown to be hypoxia-induced^36^ (Fig. 4a, b). We investigated the relationship between vernalizing conditions and anaerobic gene expression in more detail, by analyzing the expression of *ADH1* using qPCR in different genetic backgrounds. *ADH1* was expressed at much higher levels in *prt6-1* and *prt6-1 vrn2-5* compared to WT and *vrn2-5* under non-vernalizing conditions (Fig. 4c), corroborating previous studies showing ectopic accumulation of anaerobic genes in *prt6-1*^19,20^. Furthermore, *ADH1* was still induced in *vrn2-5* in response to cold treatment, and was hyper-induced in *prt6-1* and *prt6-1 vrn2-5* seedlings. This indicates that VRN2 accumulation in the cold does not significantly influence *ADH1* transcription, and that other factors, which are altered in *prt6-1*, promote anaerobic gene expression in response to cold.

Since ERFVII transcription factors are known substrates of the N-end rule pathway that induce anaerobic gene expression in response to O_2_-deprivation^19,20,26^, we examined their role in regulating cold-triggered expression of hypoxia-associated genes. Similarly to VRN2, cold temperatures led to strong stabilization of the ERFVII proteins RAP2.3-HA and HRE2-HA (Fig. 4d). Furthermore, the cold-responsive hyper-induction of *ADH1* and *HB1* in *prt6-1* was reverted in a *prt6 erfVII*^27^ sextuple mutant that lacks all 5 ERFVIIs (Fig. 4e, f and Supplementary Figure 6), suggesting that the cold-induced accumulation of ERFVIIs may control anaerobic gene induction. However, *ADH1* and *HB1* were still significantly upregulated by cold exposure in *prt6 erfVII*, albeit to a lesser extent than in WT (Fig. 4f and Supplementary Figure 6). These data suggest that upregulation of anaerobic genes in the cold could be potentiated by stabilized ERFVIIs, but that it is also controlled by a mechanism that is independent of ERFVIIs and the canonical hypoxia signalling pathway.

*PRT6* expression is not significantly altered by cold temperature (Fig. 4b), indicating that enhanced stabilization of VRN2 and ERFVIIs is not a consequence of changes in N-recognin E3 ligase levels. However, we found in vitro PCO enzyme activities towards both VRN2 and ERFVII Nt-peptides were significantly reduced at 5 °C relative to 22 °C (Fig. 4h and Supplementary Figure 4b–d). Furthermore, NO levels were significantly reduced in the roots of seedlings exposed to long-term cold conditions (Fig. 4h). Since NO-depletion can also promote accumulation of VRN2 (Fig. 2b, d) and ERFVIIs^21^, we propose that cold-exposure may induce conditions that enhance the stability of Cys-initiating N-end rule substrates, including both VRN2 and the ERFVIIs, and that this stabilization may be partly responsible for the observed overlap in gene expression.

### Angiosperm-specific recruitment of Su(z)12 to the N-end rule

**Fig. 5:**
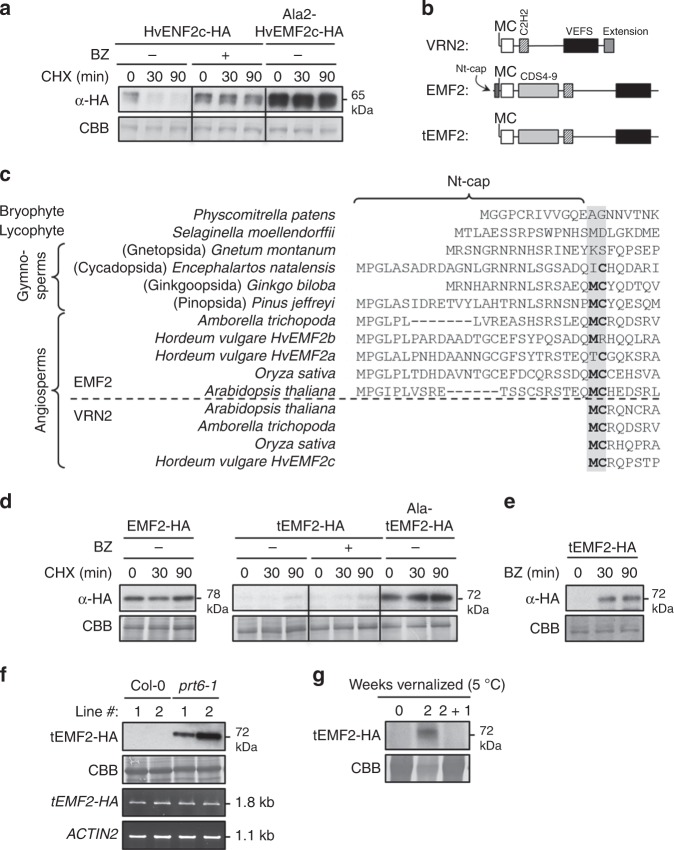
Angiosperm-specific recruitment of Su(z)12 to the N-end rule pathway. **a** In vitro cycloheximide (CHX) chase of WT and mutant (Ala2) variants of the barley VRN2-like protein HvEMF2c (±bortezomib; BZ). **b** Schematic showing the structure and key domains of *Arabidopsis* VRN2, EMF2 and truncated tEMF2. **c** Sequence alignment of the Nt of EMF2 and VRN2 proteins from land plants (above and below dashed line, respectively), showing the position of the MC dipeptide. For sequences see Supplementary Data 4. **d** In vitro CHX chase of full-length *Arabidopsis* EMF2-HA (initiating MP-), and WT or mutant (Ala2) tEMF2-HA, ±BZ. **e** In vitro accumulation over time of tEMF2-HA expressed in rabbit reticulocyte when co-incubated with BZ, but without CHX. **f** In vivo steady state protein and RNA levels of truncated WT tEMF2-HA in Col-0 vs *prt6*-1. Two independent transformants are shown. **g** Protein levels of tEMF2-HA in Col-0 during a vernalization timecourse. Source data are provided as a Source Data file

We explored the conservation and evolutionary origins of the connection between PRC2 and the N-end rule pathway. We identified and cloned an MC-initiating VRN2-like protein (HvEMF2c) from barley, a monocot species distantly related to Arabidopsis, and confirmed in vitro that it harbors a functional N-degron (Fig. 5a). However, we did not identify MC-initiating Su(z)12 homologs in basal land-plants or in animals. This suggests that O_2_-sensitive Su(z)12 proteins are only found in angiosperms, although in some species, VRN2-like proteins might evade degradation despite carrying this N-terminal sequence, similar to the SUB1A ERFVII in rice^19^.

We investigated how Su(z)12 was recruited as a substrate of the N-end rule pathway specifically in the flowering plant lineage. VRN2 is proposed to have evolved following duplication of an ancient EMF2-like Su(z)12 gene^37,38^. Alignment of Arabidopsis EMF2 and VRN2 protein sequences shows that the MC-N-terminus of VRN2 is equivalent to an internal MC-dipeptide in the N-terminal cap region of EMF2 21 amino acid residues downstream of Met1 (Fig. 5b, c and Supplementary Figure 7), that is also present in EMF2-like proteins from gymnosperm taxa, but not in more basal land plants (Fig. 5c). This dipeptide sequence occurs in the basal angiosperm *Amborella trichopoda* and many other angiosperm taxa, but has diverged in some extant angiosperm groups, suggesting that it is not important for EMF2 function (Fig. 5c and Supplementary Figure 8). Collectively these phylogenetic data suggest the internal MC dipeptide was fixed before angiosperms evolved, giving the capacity for an ancient EMF2-like protein to become an O_2_-regulated VRN2-like protein following removal of the Nt-cap. To test this, we cloned *Arabidopsis* EMF2 and showed full-length EMF2-HA was stable in vitro, but N-terminally truncated EMF2-HA (tEMF2-HA; Fig. 5b) was extremely unstable via its Cys2 residue (Fig. 5d, e). This was confirmed in planta, where tEMF2-HA could only be detected in the *prt6-1* mutant despite equivalent transgene mRNA levels in Col-0 (Fig. 5f). Moreover, this N-terminal truncation was sufficient to couple tEMF2-HA protein accumulation to cold exposure, similar to VRN2 (Fig. 5g). These data therefore support a mechanism for the evolutionary co-option of Su(z)12 to the N-end rule pathway in angiosperms, via gene duplication^37^ and N-terminal truncation of an ancient EMF2-like protein.

## Discussion

Here, we report that flowering plants have uniquely evolved a variant of the Su(z)12 PRC2 component—VRN2—that is regulated by the O_2_- and NO-sensitive branch of the Arg/N-end rule pathway. This proteolytic regulation is required to prevent ectopic accumulation of VRN2 outside of meristems in the absence of external stimuli such as cold or hypoxia, despite constitutive *VRN2* mRNA expression^38^. We show that both submergence and cold-exposure promote VRN2 accumulation. It will now be important to investigate in detail the connection between proteolytic control of VRN2 by the N-end rule pathway and the molecular changes that occur at known (e.g*. FLC*) and novel genome-wide targets of VRN2-PRC2.

Hypoxia frequently occurs in plants during development and in response to external abiotic stress such as flooding^30,39^. Reduced oxygen availability is transduced into transcriptional changes by the ERFVII transcription factors, which promote hypoxia tolerance through enhancing expression of key genes associated with anaerobiosis^19,20^. Here, we show that VRN2 stability is also regulated by O_2_, and that it could contribute to hypoxia stress-tolerance. As part of a chromatin-modifying complex, it is possible that VRN2 may be involved in the epigenetic control of gene expression linked to hypoxia acclimation, or NO signalling^40^. Together with ERFVIIs, this indicates that plants could modulate the transcriptional and epigenetic control of gene expression in response to O_2_ and NO availability by targeting functionally distinct proteins to the same proteolytic pathway, and suggests others remain to be identified^41^. Future studies examining the effect of physiological fluctuations of these gases on VRN2 accumulation, and the subsequent effect on the genome-wide targets of VRN2, may shed light on this. It is interesting to note that VIN3, another key protein that is transcriptionally induced by cold and essential for the vernalization response^35^, is also upregulated by hypoxia and may also therefore be required for VRN2 functions in response to low-O_2_.

VRN2 was originally identified as a positive regulator of vernalization, but a mechanism explaining its environmentally-induced accumulation in response to cold temperatures has not been described^14,15^. We found that long-term cold inhibits VRN2 proteolysis and that the N-end rule pathway is involved in restricting the accumulation of VRN2 in the absence of cold temperatures. Prolonged growth in the cold led not only to VRN2 accumulation, but also the induction of other molecular events that are also triggered by hypoxia, including stabilization of ERFVII transcription factors and upregulation of core anaerobic response genes. Exposure to cold temperatures has previously been shown to interfere with respiration through altering membrane fluidity properties in mitochondria, which initiates a shift to anaerobic metabolism^32,33^. Furthermore, transcriptional similarities between plants subjected to either hypoxic stress, or chemical inhibition of mitochondrial respiration, have also been reported^42^. Recently it was hypothesized that cold and hypoxia both represent an ‘energy crisis’ in plants, which may explain similar metabolic responses^43^.

Given the observed similarities in the cellular response to hypoxia and cold, we speculate that the accumulation of ERFVII transcription factors and VRN2 during cold-exposure could be related to biochemical changes that induce or mimic O_2_ limitation. We also investigated O_2_-independent mechanisms to explain cold-induced accumulation of VRN2 and ERFVIIs, and found that PCO activities and NO levels are both reduced at lower temperatures. Both of these situations could promote accumulation of O_2_-sensitive N-end rule proteins even if O_2_ is present. Nevertheless, further work is needed to show if and how the N-end rule-dependent turnover of VRN2 and ERFVIIs is modified during cold exposure.

To conclude, our work has uncovered a proteolytic mechanism operating on the PRC2 machinery that functions specifically in flowering plants. This was achieved through the lineage-specific evolution of a conditional gas-sensitive N-degron in the Su(z)12 component, perhaps representing a step toward PRC2 neo-functionalization in angiosperms. Given the key roles for VRN2 in regulating the coordination of flowering with seasonal cues, this coupling of Su(z)12 to the Arg/N-end rule pathway may have facilitated a connection between environmental conditions and PRC2 activity, whilst also permitting further diversification of PRC2 function.

## Methods

### Plant growth and materials

*A. thaliana* (Arabidopsis) seedlings were obtained from the Arabidopsis Stock Centre (NASC), except for: VRN2-FLAG^15^ (from Dr. Chris Helliwell, CSIRO, Australia), and *pFLC::FLC-GUS* (C24 ecotype, containing 6 kb FLC-GUS construct^31^, from Dr. Candice Sheldon, CSIRO, Australia). The 35S::HRE2-HA and 35S::RAP2.3-HA transgenic lines, as well as *prt6-1*, *ate1-2 ate2-1* and *prt6-1 erfVII* sextuple mutants were described previously^19,21,27,28^. Molecular characterisation of *vrn2-5* (SALK_201153) is described in Supplementary Figure 5. Mutant combinations were generated by crossing, and confirmed by PCR genotyping and RT-PCR (primers in Supplementary Table 1). Seeds were surfaced sterilized in 20% Parazone, plated on half-strength Murashige and Skoog (MS) medium (1% agar, pH 5.7, grown vertically), and stratified at 4 °C for a minimum of 2 days, before being transferred to long day (LD; 16 h L:8 h D) condition under white fluorescent light (90–100 μmol m^−2^ s^−1^) at 22 °C, and transferred to soil after 2 weeks if necessary.

### Plant phenotypic analyses

For the waterlogging assays, 12days old seedlings were transferred to soil and grown under neutral days (ND; 12 h L:12 h D). Two replica trays were set up with genotypes distributed quasi-randomly. After 12 further days of growth in soil, one tray was subjected to waterlogging for 21 days (water level maintained at the soil surface), whilst the other (control) was watered as normal with good drainage. The fresh weight of each rosette was then measured. To measure root hypoxia survival, seedlings were grown under SD conditions at 20 °C on vertical 1/2 MS plates. After 4 days of growth, seedlings were placed into sealed desiccators. 100% N_2_ was flushed through the desiccators at a flow rate of 4 L/min, until oxygen levels fell to below 0.1%. After hypoxia treatment, seedlings were returned to SD conditions for 3 further days. Roots were scored as dead if there was no growth following hypoxia treatment.

### Construction of transgenic plants

To generate C-terminally GUS-tagged VRN2 driven by its native promoter, the full genomic DNA sequence (from approx. 2 kb upstream of the ATG and ending at the STOP codon) was amplified from seedling genomic DNA extracts using attB-flanked primers, and recombined into pDONOR201 using gateway BP clonase (Invitrogen; 11789020). The C2A mutation was incorporated using *dpnI*-mediated site-directed mutagenesis, prior to mobilisation into the destination binary vector pGWB533 using LR clonase (Invitrogen; 11791100). To create 35S::tEMF2-HA (truncated EMF2) lines, tEMF2 was amplified from seedling cDNA, directionally cloned into pE2c and mobilised into pB2GW7. For all cloning primers see Table S1. Transformation into *Agrobacterium tumefaciens* (strain GV3101 pMP90) and Arabidopsis was performed according to established protocols. At least 8 independent transgenic plants were selected for each construct; data from 2–3 independent T_3_ homozygous lines are shown.

### In vivo protein stability analyses

Total protein was extracted from un-treated or treated 7-day-old (or appropriately vernalized) seedlings by grinding up frozen samples directly into SDS protein extraction buffer (125 mM Tris–HCl pH 8.8, 1% (w/v) SDS, 10% (v/v) glycerol, 50 mM Na_2_S_2_O_5_) using a micropestle, before centrifugating and determining protein concentration in the supernatant using the BioRad DC Protein Assay (Bio‐Rad)^19^. To assess the effects of submergence-induced hypoxia on in vivo protein stability, seedlings were immersed in 2 ml Eppendorf tubes filled with degassed water, sealed with parafilm, and incubated in the dark at room temperature for the times indicated. To test the effects of nitric oxide scavenging, seedlings were incubated in liquid 1/2 MS with 200 μM cPTIO for 6 h (Enzo life science; ALX-430-001)^19,21^. For cycloheximide-chase assays, seedlings were transferred to liquid 1/2 MS in 6-well microtiter plates supplemented with 100 mM cycloheximide (Sigma-Aldrich; C4859), 100 mM bortezomib (ApexBio technology; A2614), both, or appropriate solvent controls. Seedlings were then incubated at 22 °C in the light with gentle shaking, and harvested at stated time points for protein/RNA extraction (in liquid nitrogen) or GUS histochemical staining.

### Histochemical staining

For histochemical analysis of β-Glucuronidase (GUS) enzyme activity, transgenic *Arabidopsis* tissues were incubated in a buffer containing: phosphate buffer (100 mM) pH 7.0, potassium ferricyanide (2 mM), potassium ferrocyanide (2 mM), Triton X-100 (0.1% v/v) and X-Gluc solution (5-bromo-4-chloro-3-indolyl-beta-D-glucuronic acid, cyclohexylammonium salt, X-GLUC Direct) (1 mM). Samples were then incubated at 37 °C in the buffer for 4–8 h. Seedlings were cleared and fixed in 3:1 ethanol:acetic acid before mounting in Hoyer's solution (30 g gum Arabic, 200 g chloral hydrate, 20 g glycerol, 50 ml water) before imaging on a light microscope.

### In vitro stability assays

To generate protein–HA fusions driven by the T7 promoter, cDNAs were PCR amplified from Arabidopsis or barley total cDNA and directionally cloned into a modified version of the pTNT (Invitrogen) expression vector (pTNT3xHA^19^). N-terminal variations were incorporated by changing the forward primer (primers in Supplementary Table 1). Cycloheximide-chase assays were then performed using the TNT T7 Coupled Reticulocyte Lysate system (Promega; L4610)^19,44^, using 50 μM bortezomib in place of MG132. For Fig. 5e, 50 μM bortezomib was added at the beginning of the reaction and no cycloheximide was used. All assays were performed at least three times.

### Immunoblotting and relative quantification

Equal total protein amounts were resolved by SDS-PAGE, and were transferred to PVDF using a MiniTrans-Blot electrophoretic transfer cell (Bio-Rad). Membranes were probed with primary antibodies at the following dilutions: anti-HA (Sigma-Aldrich; H3663), 1:2000; anti-GUS (Sigma-Aldrich; G5420), 1:1000; anti-FLAG (Sigma-Aldrich; F1804), 1:1000. HRP-conjugated anti-mouse or rabbit secondary antibodies (Santa Cruz; sc-358914 and sc-2004) were used at a titre of 1:10,000. Immunoblots were developed to film using ECL western blotting substrate (Pierce). For quantification of steady state levels in different mutant backgrounds, relative pixel density of protein bands from three independent lines for each transgene was assessed using imageJ. All blots were performed at least three times.

### Analysing the activities of AtPCO1-5 in vitro

*A. thaliana* PCOs 1 to 5 were expressed and purified by sequential steps of immobilized nickel affinity and size exclusion chromatography as previously described^45^. The activities of each AtPCO were examined by incubating 200 µM of peptide corresponding to the first 14 amino acids of the methionine excised N-terminus of VRN2 (VRN2_2–15_) or RAP2.2/2.12 (RAP2_2–15_) with and without 0.1–0.8 µM enzyme in a bench top thermocycler (Eppendorf) at 5 or 22 °C under aerobic conditions. Time points were taken at regular intervals by quenching the reaction 1 in 10 with 1% formic acid, allowing oxidation to be monitored by ultra-high performance chromatography (UPLC)-mass spectrometry (MS). Turnover was quantified by comparing the areas underneath the product and substrate ions extracted from the total ion current chromatogram. UPLC-MS measurements were obtained using an Acquity UPLC system coupled to a Xevo G2-S Q-ToF mass spectrometer (Waters) operated in positive electrospray mode. Instrument parameters, data acquisition and data processing were controlled by Masslynx 4.1 with source conditions adjusted to maximise sensitivity and minimise fragmentation. Samples were injected on to a Chromolith Performance RP-18e 100-2 mm column (Merck) heated to 40 °C and eluted at 0.3 ml/min using a gradient of 95% deionized water supplement with 0.1% (v/v) formic acid to 95% acetonitrile.

### NO detection by fluorescence microscopy

Intracellular NO levels were visualized using DAF-FM diacetate (7′-difluorofluorescein diacetate, Bio-Connect). Seedlings were incubated in the dark for 15 min in 10 mM Tris–HCl buffer (pH 7.4) containing 50 μM DAF-FM DA and subsequently washed twice for 5 min 10 mM Tris–HCl buffer (pH 7.4). Several roots of all treatments were mounted on the same slide to allow direct comparison. Fluorescence was visualized using a Zeiss Observer Z1 LSM7 confocal microscope with excitation at 488 nm and emission 520 nm. Roots (0v) incubated and mounted in 10 mM Tris–HCl buffer (pH 7.4) without DAF-FM DA were used as a negative control to set background values. Within experiments, pinhole, gain, laser power and detector offset were identical for all slides. Mean DAF-FM DA fluorescence pixel intensity within the root was determined using ICY software (http://icy.bioimageanalysis.org/).

### Phylogenetic analyses

EMF2 and VRN2-like sequences from flowering plants (NCBI organism ID: angiosperms [taxid:3398]) were obtained using the Arabidopsis EMF2 or VRN2 protein sequence, and sequence similarity assessed using the NCBI (https://blast.ncbi.nlm.nih.gov) Protein Basic Local Alignment Search Tool (BLASTP). Protein sequences representing diverse angiosperm clades (Supplementary Data 2 and Supplementary Data 3) were aligned using MUltiple Sequence Comparison by Log-Expectation (MUSCLE)^46^ in the programme MacVector Inc. (NC, USA). Weblogo 3.0 was used to obtain a graphical representation of amino acid proportions at the amino-terminus of VRN2^47^. EMF2-like sequences from non-angiosperm plants were obtained either from genome sequences or from sequenced transcriptomes of other species via the 1kp project^48–58^ (Supplementary Data 4). Multiple sequence alignments were carried out as described above.

### Reverse transcriptase PCR and qPCR

For semi-quantitative RT–PCR, RNA was extracted from seedlings using an RNEasy plant mini kit (Qiagen; 74904) and converted to cDNA with Superscript II Reverse transcriptase (Invitrogen; 18064-014) using OligodT primers. PCRs were then performed with gene-specific or transgene-specific (i.e. gene-specific forward, tag-specific reverse) primers, and *ACTIN-2* was amplified for use as a loading control. For quantitative assessment of *FLC* expression, RNA was extracted from 11-day-old seedlings grown at 22 °C and converted to cDNA as described above. Real-time quantitative RT-PCR was performed in triplicate using Brilliant III UF MM SYBR QPCR Low ROX master mix (Agilent; 600892) on an AriaMx Real-Time PCR system (Agilent) according to manufacturer’s instructions. Relative transcript levels for *ADH1 or HB1* were determined by normalization to *ACTIN*. The control value was converted to 1 and relative fold change for other lines and treatments calculated. Data shown are mean of three biological repeats. Error bars indicate standard deviation. For primer sequences see Supplementary Table 1.

### RNA sequencing

RNA was extracted from non-vernalized (0v) or 4 weeks vernalized (4v) seedlings grown on vertical 1/2 MS plates as described above. Three biological replicates for each treatment were used for subsequent RNA sequencing, which was carried out at Glasgow Polyomics (www.polyomics.gla.ac.uk). An Initial QC was carried out using a nanodrop (to measure concentration) and the quality was tested on the Agilent Bioanalyser on a RNA Nano chip to ensure RIN values were above 8. The library was then prepared using the Illumina TruSeq mRNA kit (polyA selection), before being analysed using the Qubit and the bioanalyser HS DNA chip. Samples were pooled and sequenced on the HiSeq 4000. 150 bp reads (75 × 2 per fragment) were generated in fastq format, and aligned to the *Arabidopsis* genome (TAIR10), and then analysed using Kallisto^59^ (https://pachterlab.github.io/kallisto/about) before being processed using the R package, DESeq2^60^ (https://bioconductor.org/packages/release/bioc/html/DESeq2.html). DESeq2 was used to compare pairs of samples and generate the final set of excel spreadsheets showing fold change differences. For subsequent analyses the gene list was filtered for genes upregulated in 4v by at least 1.5 fold, with a *p* value < 0.05. Venn diagrams of overlapping gene sets were generated using Venny 2.1, and expression heatmaps created using the BAR HeatMapper tool (https://bar.utoronto.ca/ntools/cgi-bin/ntools_heatmapper.cgi). The hypergeometric test used to determine the significance of overlap measures the probability of observing a 65 or more gene overlap between two lists of length 2101 and 342 chosen at random out of 15,898 genes. 2101 is the number of cold upregulated genes that are also on the ATH1 microarray used in Gibbs et al.^19^, and 15,898 is the genes that were induced by hypoxia in Gibbs et al.^19^.

### Reporting summary

Further information on experimental design is available in the Nature Research Reporting Summary linked to this article.

## Supplementary information

Supplementary Information

Description of Additional Supplementary Files

Supplementary Data 1

Supplementary Data 2

Supplementary Data 3

Supplementary Data 4

Reporting Summary

Source Data File

## Data Availability

RNA-seq data is available at NCBI GEO database with accession code GSE123459. The source data underlying all figures can be found in the Source Data file.
