# Supplementary material for: Oxygen-dependent proteolysis regulates the stability of angiosperm polycomb repressive complex 2 subunit VERNALIZATION 2
Source: Nat Commun. 2018 Dec 21;9:5438. doi: 10.1038/s41467-018-07875-7 (PMC6303374; doi:10.1038/s41467-018-07875-7)
Supplement: Supplementary file 1 — Supplementary Information [file 41467_2018_7875_MOESM1_ESM.pdf]

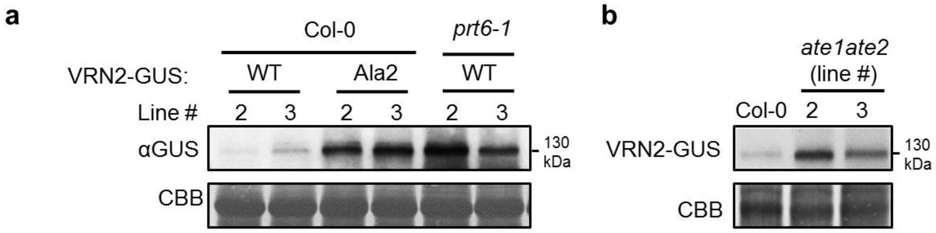

Supplementary Figure 1. Supplemental western blots. (**A, B**) Steady state protein levels of WT and Ala2-VRN2-GUS in Col-0, *prt6-1* and *ate1 ate2*. Two independent lines are shown, in addition to a third line in Fig. 1e and f. These independent lines were used to calculate average relative densities in Fig 1g. Source Data are provided as a Source Data file.

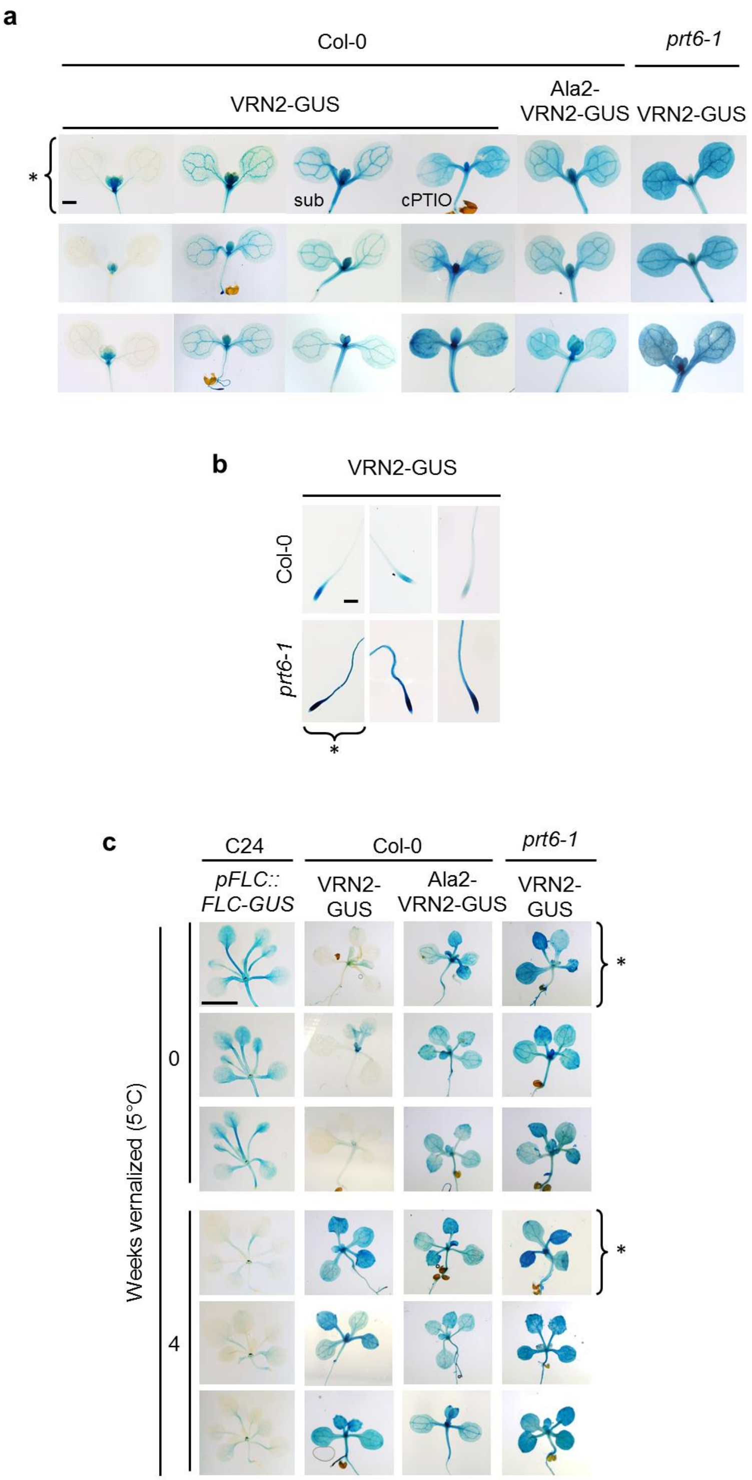

Supplementary Figure 2. Biological replicates of histochemical GUS stains. (**A**) Replicates of data presented in Figs. 1i and 2: 7-day-old Col-0 or *prt6-1* seedlings expressing WT or mutant

(Ala2) VRN2-GUS +/- BZ, submergence or cPTIO treatment. (**B**) Replicates of data presented in Fig 1j: 7-day-old primary root tips of Col-0 or *prt6-1* expressing WT VRN2-GUS. (**C**) Replicates of data presented in Fig. 3b: Seedlings expressing *pFLC::FLC-GUS* (C24 background), and WT or mutant (Ala2) VRN2-GUS in Col-0 and *prt6-1*, +/- 4 weeks vernalization. Asterisks refer to the rows or columns showing images that are replicated in the corresponding main figure.

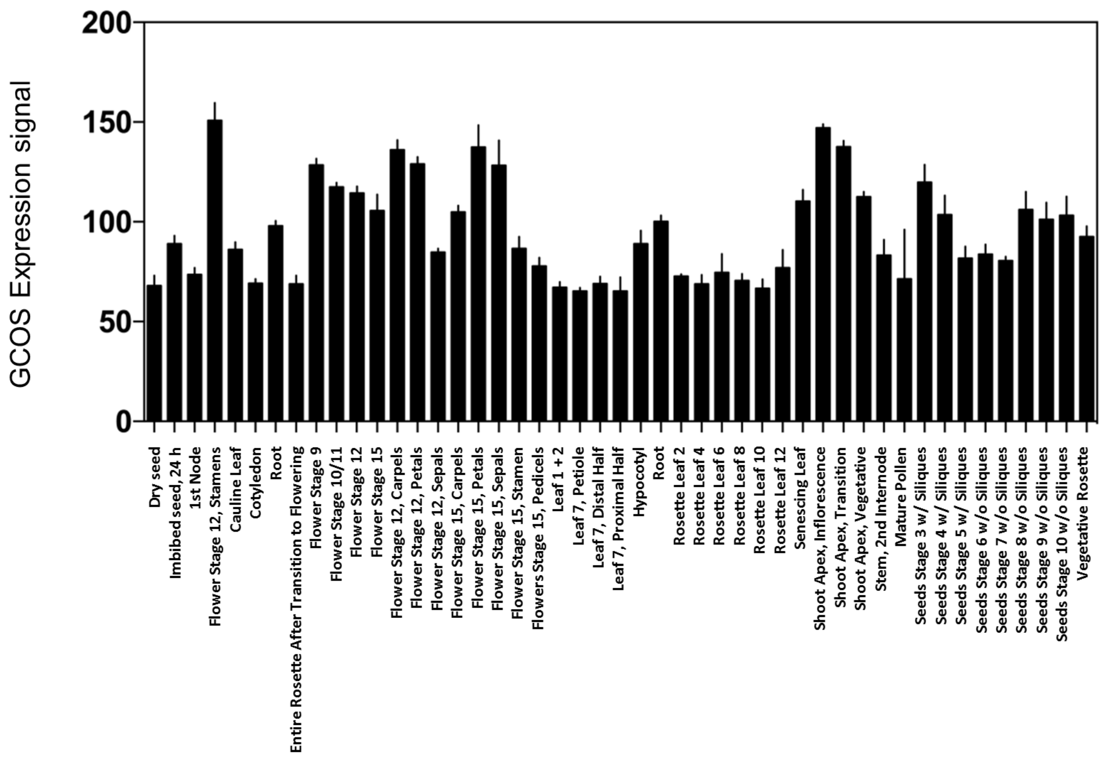

Supplementary Figure 3. *VRN2* mRNA expression levels in diverse tissues of Arabidopsis throughout development, obtained from the eFP browser (http://bbc.botany.utoronto.ca/efp/cgi-bin/efpWeb.cgi?dataSource=Developmental_Map)

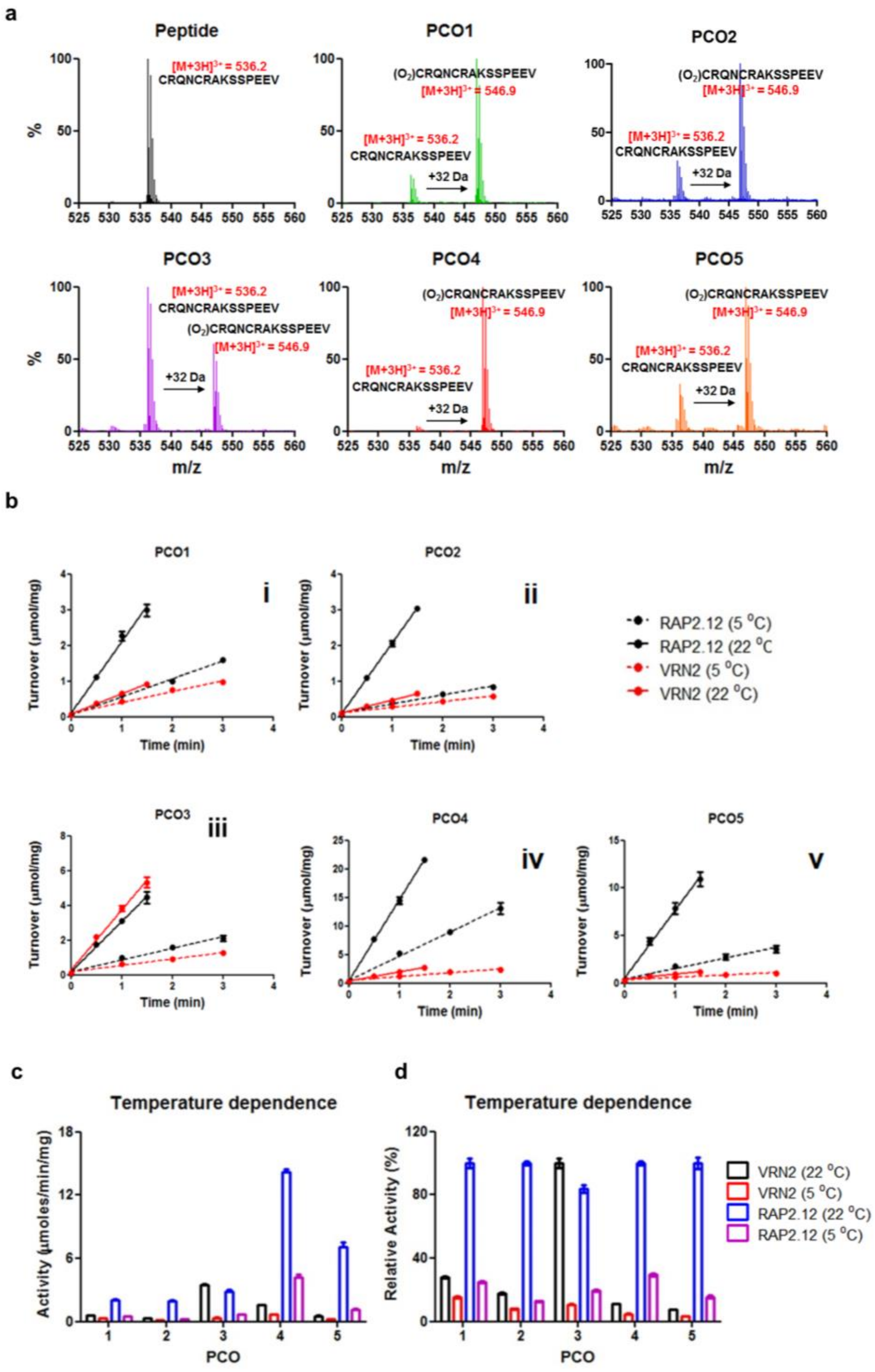

Supplementary Figure 4. PCO activity at the N-terminus of VRN2. (**A**) Mass spectra of a peptide corresponding to the methionine excised N-terminus of VRN2 following incubation with (colour) and without (black) AtPCO1-5. Clear masses consistent with Nt-Cys sulfinic acid

formation, the oxidised product of PCO turnover, could only be observed in the presence of PCO enzymes. Data presented here for peptide-only and PCO1 are the same as used in Fig. 1k (**B**) Rate profiles showing the turnover of a peptide corresponding to the methionine excised N-terminus of RAP2.2/2.12 (black) and VRN2 (red) with AtPCO1 (i), 2 (ii), 3 (iii), 4 (iv) and 5 (v) over time at 5 ^{o}C (dashed) and 22 ^{o}C (solid), which were used to calculate the specific activities presented in **C**. Activity was subsequently normalised relative to the highest value obtained for each PCO to eliminate variation between different homologs. n=3 for each PCO-peptide combination. (**D**). Rate of PCO turnover was observed to decrease at the lower temperature for both substrates, although fold reduction varied between homolog, RAP2.2/2.12 and VRN2**.** Source Data are provided as a Source Data file.

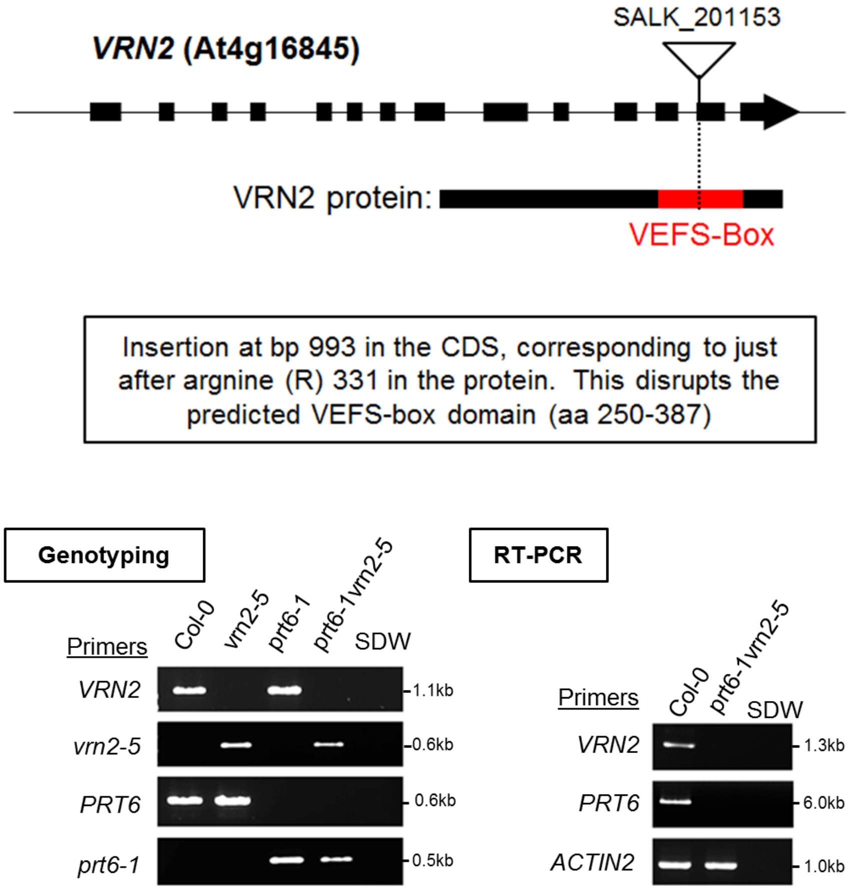

Supplementary Figure 5. Genotyping of new mutant lines and combinations used in this study. Schematic shows location of *vrn2-5* T-DNA insertion in the VRN2 gene and protein. VEFS box = (VRN2-EMF2-FIS2-Su[z]12) box, a critical functional domain found in Su(z)12 proteins. Genotyping data confirming homozygosity for combination mutants is shown, as is RT-PCR confirming knock out of full length mRNA.

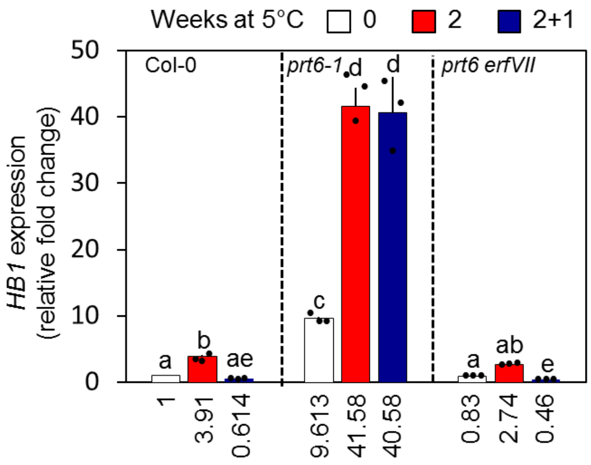

Supplementary Figure 6. qPCR of *HB1* levels in Col-0, *prt6-1* and *prt6 erfVII* seedlings exposed to 5°C for 0 or 2 weeks, or 2 weeks followed by 1 week at 22C (2+1). Each bar shows the mean of 3 biological reps (dots). Expression values are shown underneath and letters indicate one-way ANOVA; Tukey's test ($P<0.05$). Data are average of 3 biological reps. Source Data are provided as a Source Data file.

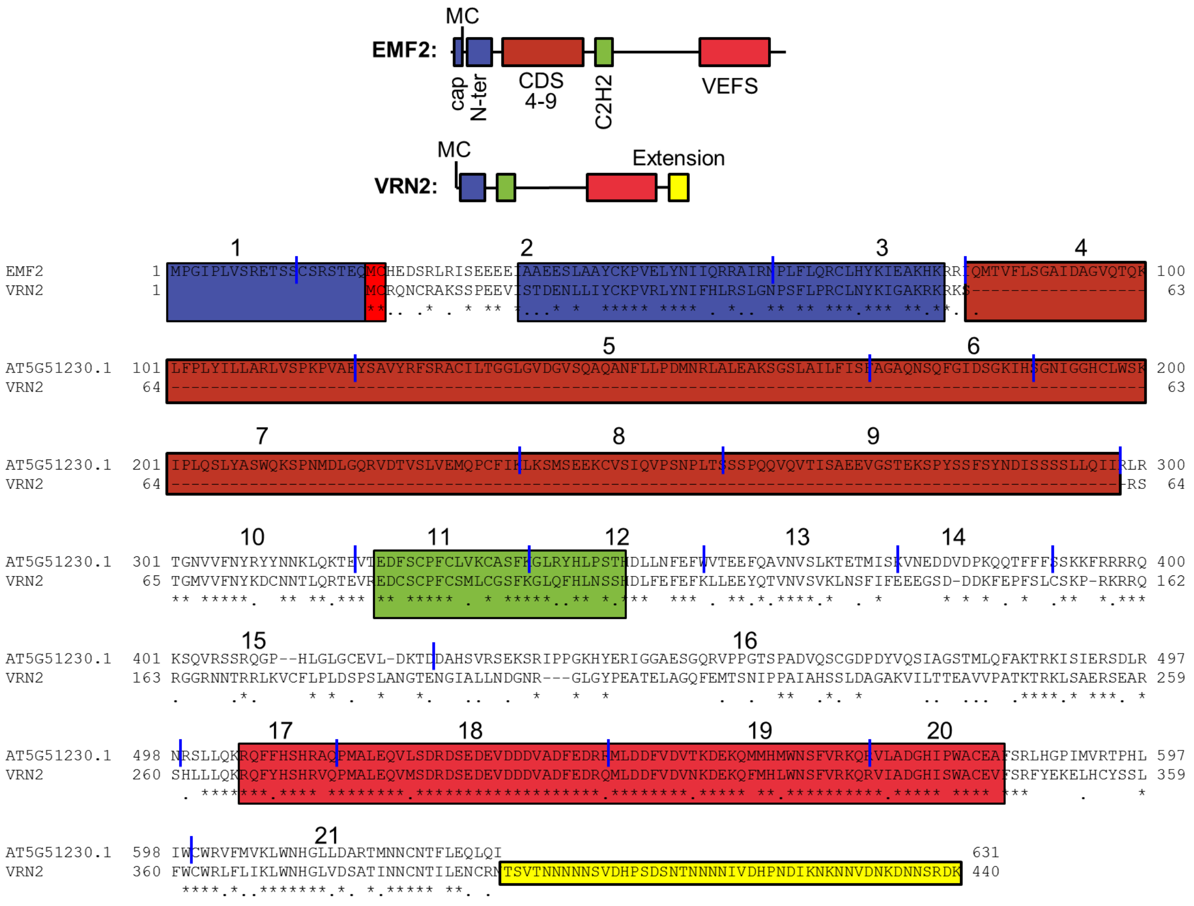

Supplementary Figure 7. Comparison of exon positions in VRN2 and EMF2, with important domains highlighted. Counting relates to the coding sequence (CDS), not exons (first exon is UTR). Positions of domains C2H2 and VEFS taken from uniprot. See also Supplementary Dataset 4.

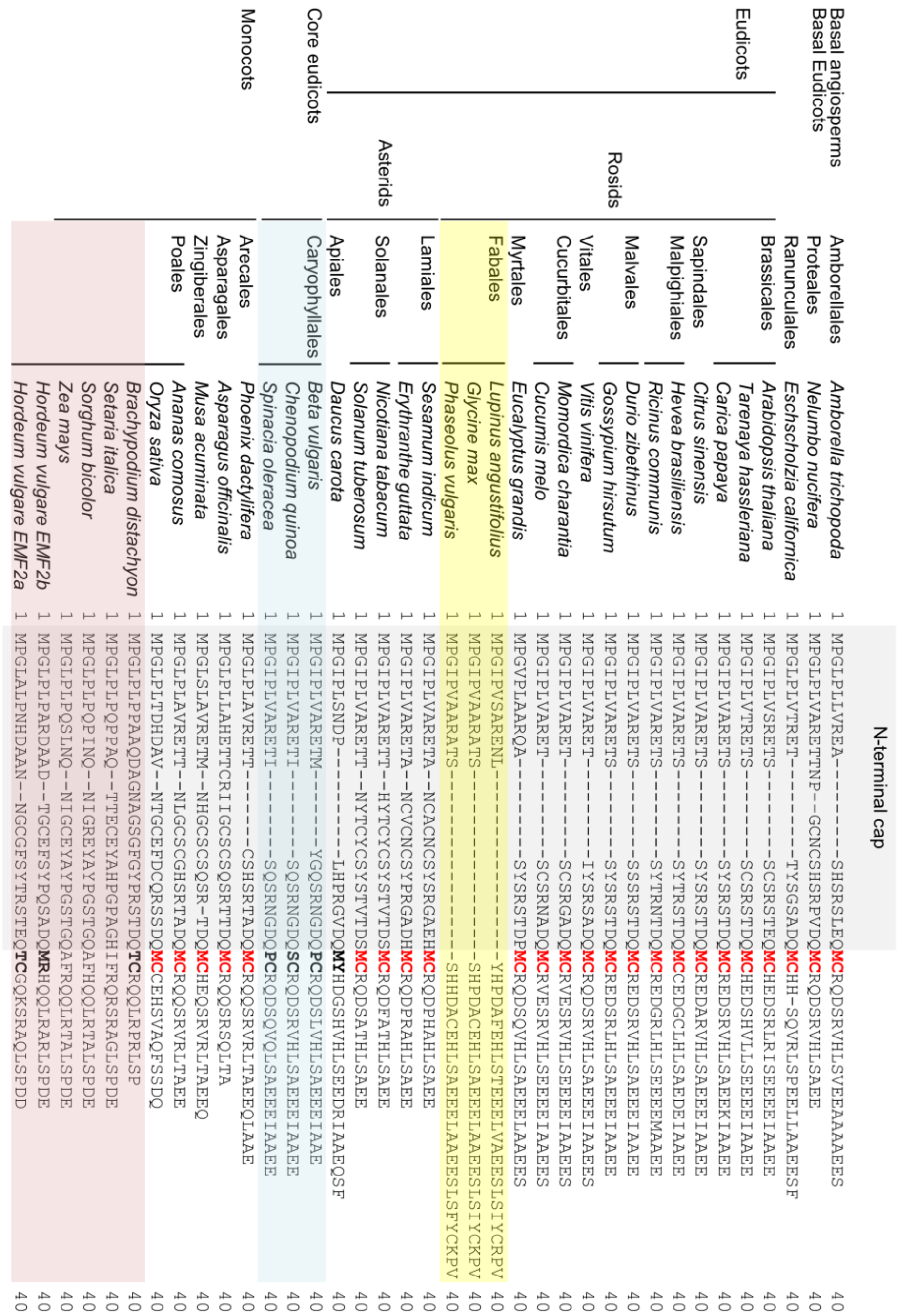

Supplementary Figure 8. Alignment of EMF2 Nt-sequences from diverse taxa showing divergence of the internal MC dipeptide at the C-terminal end of the Nt-cap domain (for full sequence details see Supplementary Dataset 3)

Supplementary Table 1. List of oligonucleotide primers

Primer name	Gene ID	sequence
Primers for RT-PCR		
VRN2 F	At4g16845	ATGTGTAGGCAGAATTGTCGCGC
VRN2 R	At4g16845	TTACTTGTCTCTGCTGTTATTGTCC
GUS fusion R	-	TGGCACAGCAATTGCCCGGC
FLAG fusion R	-	CTTATCGTCGTCATCCTTGTAATC
VIN3 F	At5g57380	GCTTCGCTCTCAAAGATCTGGC
VIN3 R	At5g57380	CCAAAGCTTGAGGCAGATCCC
PRT6 F	At5g02310	GTTTGCTACAGGCTACAAAGT
PRT6 R	At5g02310	GGTCAAGAGTGTCAACCATGATC
ACTIN2 F	At3g18780	ATGGCTGAGGCTGATGATATTC
ACTIN2 R	At3g18780	AGAAACATTTTCTGTGAACGATTC
ADH1 F	At1g77120	TCTACCACCGGACAGATTATTCG
ADH1 R	At1g77120	TCAAGCACCCATGGTGATGATGC
HB1 F	At2g16060	ATGGAGAGTGAAGGAAAGATTGTG
HB1 R	At2g16060	TTAGTTGGAAAGATTCATTTCAGC
PDC1 F	At4g33070	GGACACCAAAATCGGATCGAT C
PDC1 R	At4g33070	CTACTGAGGATTGGGAGGACG
SUS4 F	At3g43190	AACGCAGAACGTGTAATAACG
SUS4 R	At3g43190	CTCGGAGTGATGTTGAGTCC
PCO1 F	At5g15120	TGGGGTTTGAGATGAAACCAG
PCO1 R	At5g15120	TCAATCTTCAACCTTTGGGCC
LBD41 F	At3g02550	ATGTCGGAGAGACCAAGCCG
LBD41 R	At3g02550	TTAATTAGAAGAGGCGTTCCC
HUP7 F	At1g43800	GCTTGCGCACAAGTCTCTTC
HUP7 R	At1g43800	TTACACACTAATCTGCTTATCG
HUP40 F	At4g24110	TGTAGGTCGCCCGATTTCG
HUP40 R	At4g24110	AGAAGAAGGTTCTTCTTGTCC
tEMF2 F	At5g51230	ATGTGCCATGAAGACTCCCGTC
3xHA R	-	TTAGTCGGGCACGTCGTAGGG
Primers for qPCR		
qPCR FLC F	At5g10140	AGCCAAGAAGACCGAACTCA
qPCR FLC R	At5g10140	TTTGTCCAGCAGGTGACATC
qPCR ADH1 F	At1g77120	GGTCTTGGTGCTGTTGGTTT
qPCR ADH1 R	At1g77120	CTCAGCGATCACCTGTTGAA
qPCR ACTIN2 F	At3g18780	TCGTACAACCGGTATTGTGCTG
qPCR ACTIN2 R	At3g18780	TTACAATTTCCCGCTCTGCTG

Primers for genotyping		
vrn2-5(SALK)LP	-	GTTTGTTCATCATGACACCCC
vrn2-5(SALK)RP	-	TTTGAGTCACTGGGATGATCC
SALK LBb1.3	-	ATTTTGCCGATTTCGGAAC
prt6-1 LP	-	GGAGTTTTCTATGTCCAGTGAGAGTTT
prt6-1 RP	-	GTCTCCAATGACACGTTCACTTGTCT
prt6-1 BP		GCCTTTTCAGAAATGGATAAATAGCCTTGCTTCC
Primers for cloning pVRN2::(MC/MA)VRN2-GUS		
pVRN2 F attB	At4g16845	GGGGACAAGTTTGTACAAAAAAGCAGGCTGTTCCAAAAGCAACATGTTG
VRN2 R attB	At4g16845	GGGGACCACTTTGTACAAGAAAGCTGGGTCCTTGTCTCTGCTGTTATTGTC
VRN2 SDM F	At4g16845	CAAGAATGGCTAGGCAGAATTG
VRN2 SDM R	At4g16845	CAATTCTGCCTAGCCATTCTTG
Primers for cloning 35S::tEMF2-HA		
tEMFc F	At5g51230	AAAAGTCGACATGTGCCATGAAGACTCCCGTC
EMF2 R	At5g51230	AAAAGCGGCCGCAATTTGGAGCTGTTCGAGAAAGG
Primers for cloning in vitro expression constructs		
HvEMF2c F	AB085819.1	AAAAGAATTCATGTGCCGTCAACCGTCCACGCC
C2A-HvEMF2c F	AB085819.1	AAAAGAATTCATGGCCCGTCAACCGTCCACGCC
HvEMF2c R	AB085819.1	AAAAGGTACCGTACTGTCGGCGGTGTAAGATTGC
EMF2 F	At5g51230	AAAAGAATTCATGCCAGGCATTCCTCTTGTTAG
tEMFc F	At5g51230	AAAAGAATTCATGTGCCATGAAGACTCCCGTC
C2A-tEMF2 F	At5g51230	AAAAGAATTCATGGCCCATGAAGACTCCCG
EMF2 R	At5g51230	AAAAGGTACCAATTTGGAGCTGTTCGAGAAAGG
PCO cloning primers:		
PCO1 pET28 F	At5g15120	CGCGCGGCAGCCATATGGGGTTTGAGATGAA
PCO1 pET28 R	At5g15120	GGTGGTGGTGCTCGAGTCAATCTTCAACCTTTGG
PCO2 pET28 F	At5g39890	CGCGCGGCAGCCATATGGGAACTGATACAGTTATG
PCO2 pET28 R	At5g39890	GGTGGTGGTGCTCGAGTCATTCTTTGATGGTTGG
PCO3 pET28 F	At1g18490	CGCGCGGCAGCCATATGTTGTCGAGATTGTTCA
PCO3 pET28 R	At1g18490	GGTGGTGGTGCTCGAGCTAGACTCTGATAGTTGGACCA
PCO4 pET28 F	At2g42670	CGCGCGGCAGCCATATGCCTTACTTTGCTCA
PCO4 pET28 R	At2g42670	GGTGGTGGTGCTCGAGTCAAGTTCTAATGACAGGACC
PCO5 pET28 F	At3g58670	CGCGCGGCAGCCATATGCCATATTTTATTCAGAGG
PCO5 pET28 R	At3g58670	GGTGGTGGTGCTCGAGTCATTTTCTAATCACTGGACC
