# Supplementary material for: Oxygen-dependent proteolysis regulates the stability of angiosperm polycomb repressive complex 2 subunit VERNALIZATION 2
Source: Nat Commun. 2018 Dec 21;9:5438. doi: 10.1038/s41467-018-07875-7 (PMC6303374; doi:10.1038/s41467-018-07875-7)
Supplement: Supplementary file 2 — Description of Additional Supplementary Files [file 41467_2018_7875_MOESM2_ESM.pdf]

Description of Additional Supplementary Files

File Name: Supplementary Data 1
Description: Differentially expressed gene lists

File Name: Supplementary Data 2
Description: VRN2 orthologous sequences representing diverse clades of flowering plants

File Name: Supplementary Data 3
Description: EMF2 orthologous sequences representing diverse clades of flowering plants.

File Name: Supplementary Data 4
Description: EMF2 and VRN2 orthologous sequences representing diverse clades of land plants
